# How to choose proper local treatment in men aged ≥75 years with cT2 localized prostate cancer?

**DOI:** 10.1002/cam4.2221

**Published:** 2019-05-08

**Authors:** Kun Jin, Shi Qiu, Jiakun Li, Xiaonan Zheng, Xiang Tu, Xinyang Liao, Yan Yang, Lu Yang, Qiang Wei

**Affiliations:** ^1^ Department of Urology, Institute of Urology West China Hospital, Sichuan University Chengdu China; ^2^ Center of Biomedical big data West China Hospital, Sichuan University Chengdu China; ^3^ Department of Nephrology, The First People's Hospital of Changzhou The Third Affiliated Hospital of Soochow University Changzhou China

**Keywords:** cancer‐specific mortality, localized prostate cancer, radiation therapy, radical prostatectomy, the elderly

## Abstract

**Background:**

For localized prostate cancer (PCa), radical prostatectomy (RP) and radiotherapy (RT) are two standard interventions to decrease PCa mortality. Contemporary studies contained the elderly people; analyses focusing on patients over 75 years of age were still lacking.

**Method:**

In the Surveillance Epidemiology and End Results (SEER) database (2004‐2015), people over 75 years of age with cT2 stage were selected in our research. Multivariable Cox proportional hazard models were used to analyze cancer‐specific mortality (CSM) and overall mortality (OM) after adjustment. The propensity score matching was performed to assume the randomization. An instrument variate (IVA) was used to calculate the unmeasured confounders.

**Results:**

Radical prostatectomy is superior to RT in OM and CSM after adjustment for covariates (HR = 0.54, 95% CI = 0.47‐0.62, *P* < 0.001 and HR = 0.30, 95% CI = 0.20‐0.45, *P* < 0.001, respectively). The cox model after matching indicated similar consequence (OM: HR = 0.53, 95% CI = 0.46‐0.62, *P* < 0.001; CSM: HR = 0.27, 95% CI = 0.17‐0.43, *P* < 0.001). In the IVA‐adjusted model, the effect of treatment changed slightly (OM: HR = 0.65, 95% CI = 0.54‐0.78, *P* < 0.001; CSM: HR = 0.21, 95% CI = 0.12‐0.37, *P* < 0.001). Subgroup analyses showed that for patients with GS = 7, those received RP obtained the highest risk decline for overall death (HR = 0.41, 95% CI = 0.32‐0.52); and for patients with younger age, those received RP obtained the highest risk decline for CSM (HR = 0.11, 95% CI = 0.01‐0.52).

**Conclusion:**

Patients over 75 years of age with cT2 stage will obtain more benefit from RP compared with RT, especially for patients with GS = 7 and younger age.

## 1 INTRODUCTION

Prostate cancer (PCa) is the second most common cancer in males, with an estimated 1.1 million confirmed cases worldwide in 2012, making up 15% deaths of all cancers diagnosed.^1^ This disease primarily encroaches on the elderly with age‐related increasing incidence rates.^2^ Autopsy studies also suggest that high‐grade intraepithelial neoplasia, the precursor of incidental PCa, are detected more easily among the old. Another European research showed that for men between 30 and 69 years of age, the prevalence of incidental PCa was 30% and sharply increased to 75% for those over 70 years of age.^3^ With the aging of population and the improvement of life expectancy, elderly people with PCa should be paid more concern.

For localized PCa, radical prostatectomy (RP) and radiotherapy (RT) are two standard interventions to decrease PCa mortality.^4^, ^5^ There is still controversy about the choice of treatment. Recently, the first randomized clinical trial, Prostate Testing for Cancer and Treatment, comparing RP, RT, and active monitoring showed that there was no significant difference of 10‐year cancer‐specific‐survival (CSS) in RP versus RT.^6^ However, several studies held the opposite opinion. Three retrospective studies indicated that people received RP was superior to RT in terms of biochemical recurrence, metastasis‐free survival, and CSS.^7^, ^8^, ^9^ These studies contained patients of all T stages and none of these focused on the elderly.

In consideration of worse physical conditions, advanced stage and higher pathological grade compared with the younger,^10^ the outcome of RP or RT among old people may differ from others. Two observational studies discussing the benefit of local treatment (RP or RT) for men ≥70 years of age of low risk and for men ≥75 years of age were performed.^11^, ^12^ Despite adjustment for covariates to control measured confounders, there remains residual confounding comparing RT with RP. In view of this, we aimed to provide risk estimates to inform contemporary treatment decision for PCa men aged ≥75 years.

## 2 METHOD

### 2.1 Study populations

**Figure 1:**
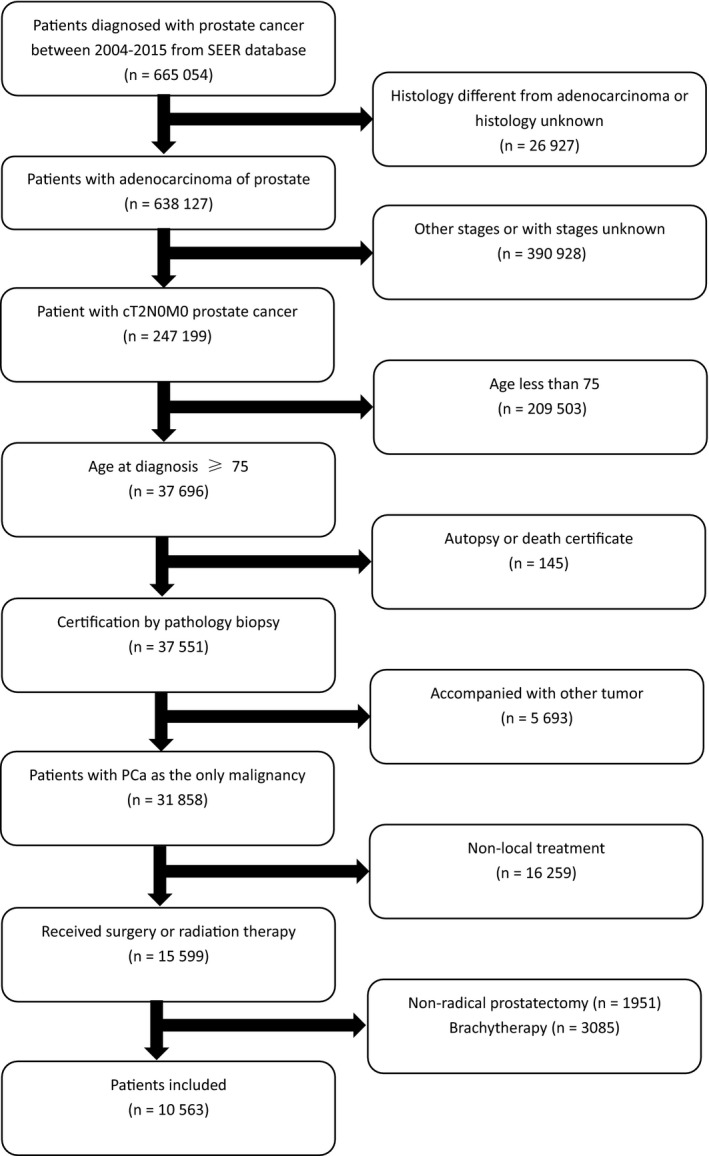
Flowchart of the patients selection

In the Surveillance Epidemiology and End Results (SEER) database (2004‐2015), old men diagnosed with histological adenocarcinoma of the prostate [International Classification of Disease for Oncology (ICD‐O‐3) code 8140 of the prostate (site code C61.9)] over 75 years of age were selected in our research. Old people with T1 stage PCa mostly received RT, causing huge bias in the analysis. As a result, our research only included individuals with T2 stage. Further inclusion/exclusion criteria are depicted in Figure 1. Our study consisted of 10 563 patients in final, who were stratified based on different therapy type: RT group versus RP group. The total available covariates were listed in Table 1.

**Table 1:** Baseline characteristics of 10 563 patients who received RT versus RP

	RT (N = 8447)	RP (N = 2116)	*P* value
Age	78.593 ± 3.184	76.894 ± 2.511	<0.001
PSA	116.632 ± 137.769	74.947 ± 88.731	<0.001
Marital status			<0.001
Married	5878 (69.587%)	1650 (77.977%)	
Single	443 (5.244%)	105 (4.962%)	
Divorced/widowed	1265 (14.976%)	252 (11.909%)	
Unknown	861 (10.193%)	109 (5.151%)	
Year of diagnosis			<0.001
2004	851 (10.075%)	163 (7.703%)	
2005	798 (9.447%)	131 (6.191%)	
2006	821 (9.719%)	155 (7.325%)	
2007	851 (10.075%)	200 (9.452%)	
2008	753 (8.914%)	161 (7.609%)	
2009	725 (8.583%)	188 (8.885%)	
2010	742 (8.784%)	221 (10.444%)	
2011	729 (8.630%)	212 (10.019%)	
2012	587 (6.949%)	158 (7.467%)	
2013	514 (6.085%)	186 (8.790%)	
2014	505 (5.978%)	154 (7.278%)	
2015	571 (6.760%)	187 (8.837%)	
Race			<0.001
White	7014 (83.035%)	1834 (86.673%)	
Black	661 (7.825%)	111 (5.246%)	
Other	618 (7.316%)	153 (7.231%)	
Unknown	154 (1.823%)	18 (0.851%)	
GS			<0.001
≤6	1195 (14.147%)	343 (16.210%)	
7	2009 (23.784%)	513 (24.244%)	
≥8	1324 (15.674%)	111 (5.246%)	
Unknown	3919 (46.395%)	1149 (54.301%)	
Region			<0.001
East	2696 (31.917%)	444 (20.983%)	
Pacific	4233 (50.112%)	1387 (65.548%)	
North	1216 (14.396%)	133 (6.285%)	
Other(Alaska and Southwest)	302 (3.575%)	152 (7.183%)	

Abbreviations: GS, Gleason score; PSA, prostate specific antigen; RP, radical prostatectomy; RT, radiation therapy.

### 2.2 Endpoint

Our main endpoint included: cancer‐specific mortality (CSM) defined as death caused by prostate malignancy (SEER code 28010); overall mortality (OM) defined as deaths from any reason reported by the SEER database. Survival time was defined as the duration from initial diagnosis to death from any cause or last follow‐up.

### 2.3 Statistical analysis

Firstly, we assessed the distribution of baseline characteristics with the use of a two‐sample t test and a chi‐square test to compare continuous and categorical variables, respectively. Data were presented as mean ± SD for continuous variables and as frequency (%) for categorical variables.

Secondly, a multivariable Cox proportional hazard model was used for analyses of OM and CSM after adjusting race, age, marital status, Gleason Score (GS), and prostate specific antigen (PSA).^13^

Thirdly, in consideration of baseline characteristics affecting the option of using different treatment methods, propensity score matching (PSM) (ratio 1:1, with a caliper set of 0.05) was performed to ensure that both the RT group and the RP group had similar baseline characteristics with the use of logistic regression to adjust for between‐group differences.^14^ The matching was conducted based on nearest‐neighbor matching principle. The matched process was considered as balanced with a *P* value >0.05.

Fourthly, considering the selection bias between patients who received RT versus RP, we additionally used an instrument variate (IVA) to calculate the unmeasured confounders. We selected yearly regional utilization rate as IVA to perform a two‐stage residual inclusion analysis.^15^, ^16^ This IVA was previously used in the literature 17, 18, 19, 20 and calculated for each of the four American regions as follows:

$$\frac{\text{RP cases/region/yr}}{\left(\text{RP cases/region/yr}\right)+\left(\text{RT cases/region/yr}\right)}$$

We calculated the F‐statistic to confirm its correlation with the option of therapy. And the residual, defined as the observed minus the predicted probability of receiving RP, was calculated. The second IVA assumption was verified that without the use of IVA, the correlation between exposure and outcome cannot be formally tested. Another multivariate Cox proportional hazard model including all covariates and residual was presented.

Sensitivity analyses were performed: (a) The analysis of CSM after adjusting propensity scores; (b) two additional models with inverse probability of treatment weighing (IPTW) and standardized mortality ratio weighting (SMRW) using the propensity score to evaluate the relationship between treatment types and outcomes including all eligible patients (entire cohort); (c) the analysis of CSM stratified by propensity scores; and (d) Competing‐risks regression assessing the CSM.

All analyses were performed with the statistical software packages R (http://www.R-project.org, The R Foundation) and EmpowerStats (http://www.empowerstats.com, X&Y Solutions, Inc, Boston, MA). A *P* value <0.05 was considered statistically significant.

## 3 RESULTS

**Figure 2:**
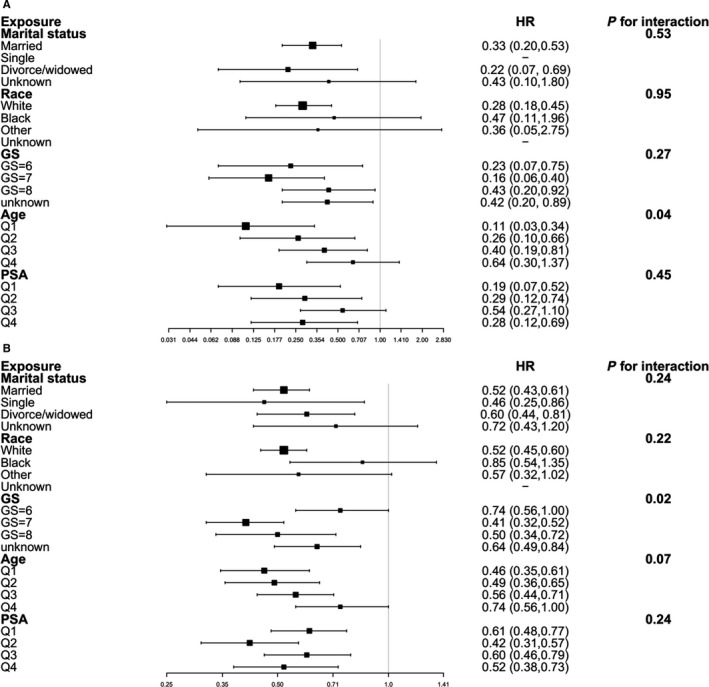
Subgroup analyses of CSM and OM (RT vs RP)

A total of 10,563 elderly men (age ≥75 years) were identified in our study; 8447 patients received RT while 2116 received RP. Details of the baseline characteristics stratified according to RT versus RP were reported in Table 1. The two treatment groups differed significantly in regard to most clinical indicators. Results of the multivariate Cox proportional hazard regression showed that RP could reduce more risks of OM and CSM after adjusted for race, age, marital status, GS and PSA (hazard ratio [HR]=0.54, 95% CI = 0.47‐0.62, *P* < 0.001 and HR = 0.30, 95% CI = 0.20‐0.45, *P* < 0.001, respectively). From the Kaplan‐Meier survival curve, the significant survival benefits were observed in the comparison of RP and RT (Figure 2).

Following PSM, there were 2152 individuals in each treatment group (Table 2). The cox model after matching indicated that RP was still superior to RT (OM: HR = 0.53, 95% CI = 0.46‐0.62, *P* < 0.001; CSM: HR = 0.27, 95% CI = 0.17‐0.43, *P* < 0.001) (Table 3). In consideration of the differences after the matching, another cox model adjusted for propensity scores was conducted (Supplement Table S2). Moreover, the results of the sensitivity analyses showed similar outcome (Supplement Table S3). In the IVA‐adjusted model, the effect of treatment changed slightly (OM: HR = 0.65, 95% CI = 0.54‐0.78, *P* < 0.001; CSM: HR = 0.21, 95% CI = 0.12‐0.37, *P* < 0.001).

**Table 2:** Baseline characteristics of 4304 patients after propensity score match according to treatment

	RT (N = 2152)	RP (N = 2152)	*P* value
Age	76.88 ± 2.30	76.89 ± 2.51	0.8791
PSA	79.51 ± 92.88	75.11 ± 88.92	0.1121
Year of diagnosis			<0.0001
2004	226 (10.50%)	165 (7.67%)	
2005	186 (8.60%)	132 (6.13%)	
2006	189 (8.78%)	156 (7.25)	
2007	195 (9.06%)	205 (9.53%)	
2008	204 (9.48%)	166 (7.71%)	
2009	189 (8.78%)	190 (8.83%)	
2010	200 (9.29%)	222 (10.32%)	
2011	200 (9.29%)	215 (9.99)	
2012	139 (6.46%)	161 (7.48%)	
2013	148 (6.88%)	193 (8.97%)	
2014	127 (5.90%)	158 (7.34%)	
2015	149 (6.92%)	189 (8.78%)	
Marital status			0.0604
Married	1728 (80.3%)	1679 (78%)	
Single	103 (4.8%)	106 (4.9%)	
Divorced/widowed	202 (9.4%)	256 (11.9%)	
Unknown	119 (5.5%)	111 (5.2%)	
Race			0.0083
White	1892 (87.9%)	1867 (86.8%)	
Black	137 (6.4%)	113 (5.3%)	
Other	105 (4.9%)	154 (7.2%)	
Unknown	18 (0.8%)	18 (0.8%)	
GS			<0.0001
≤6	324 (15.1%)	346 (16.1%)	
7	487 (22.6%)	522 (24.3%)	
≥8	277 (12.9%)	115 (5.3%)	
Unknown	1064 (49.4%)	1169 (54.3%)	
Region			<0.0001
East	744 (34.6%)	452 (21%)	
Pacific	1006 (46.7%)	1413 (65.7%)	
North	319 (14.8%)	134 (6.2%)	
Other(Alaska and Southwest)	83 (3.9%)	153 (7.1%)	

Abbreviations: GS, Gleason score; PSA, prostate specific antigen; RT, radiation therapy; RP, radical prostatectomy.

**Table 3:** Multivariate cox regression analyses for OS and CSM in the total cohort and matched population

Outcome	Treatment	Non‐adjusted model	Adjusted model	PSM model	IVA‐adjusted model
OM	RT	1.0	1.0	1.0	1.0
	RP	0.42 (0.37, 0.48)	0.54 (0.47, 0.62)	0.53 (0.46, 0.62)	0.65 (0.54, 0.78)
CSM	RT	1.0	1.0	1.0	1.0
	RP	0.21 (0.14, 0.32)	0.30 (0.20, 0.45)	0.27 (0.17, 0.43)	0.21 (0.12, 0.37)

Note:

Adjusted model: adjusted for race, age, marital status, Gleason score (GS) and prostate specific antigen (PSA). Propensity score matching (PSM) model: matched according to race, age, marital status, GS and PSA. Instrument variate (IV) adjusted model: adjusted for race, age, marital status, GS and PSA and residual

Abbreviations: CSM, cancer specific mortality; OM, overall mortality.

**Figure 3:**
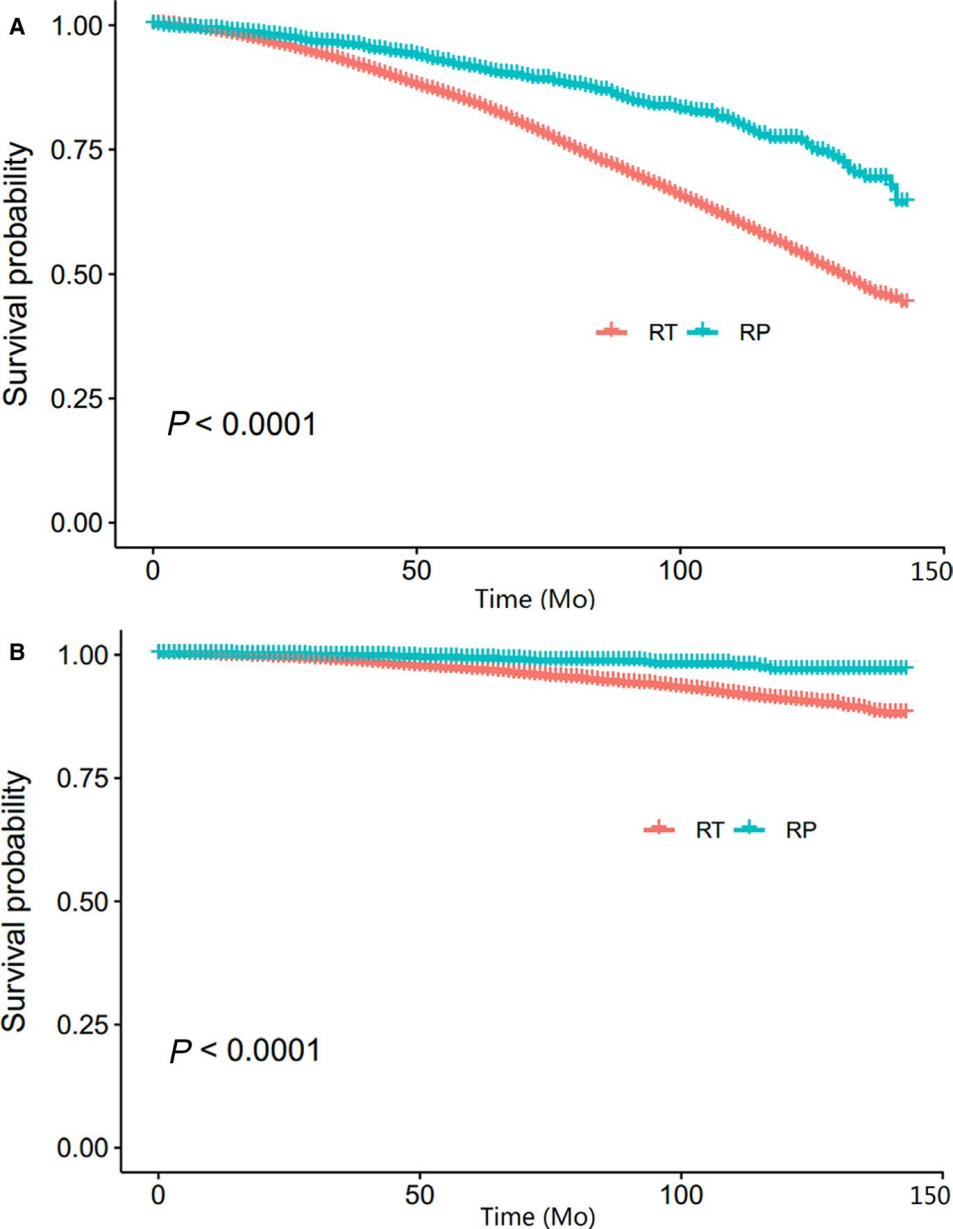
Kaplan‐Meier survival curve of CSM and OM (RT vs RP)

In the subgroup analyses (Figure 3), no significant interaction was observed between the effect of OS and marital status or race (P value of interaction, 0.24 and 0.22, respectively). We did observe a larger magnitude of association between GS and OS (P value for interaction, 0.02). The results indicated that for patients with GS = 7, those received RP obtained the highest risk decline for overall death (HR = 0.41, 95% CI = 0.32‐0.52). And no significant interaction was observed between the effect of OS and age or PSA (*P* value of interaction, 0.07 and 0.24, respectively). In the subgroup analyses of CSM, a significant relationship between CSM and age was observed (P value for interaction, 0.02), indicating that for patients with younger age, those received RP obtained the highest risk decline for CSM (HR = 0.11, 95% CI = 0.01‐0.52). Other covariates such as marital status, race, GS, and PSA showed no significant interaction effects (*P* value of interaction, 0.53, 0.95, 0.27 and 0.45, respectively).

## 4 DISCUSSION

Among PCa people with the age ≥75, it still remains uncertain regarding the efficacy of modalities of local treatment. Several studies focusing on the role of RT or RP have excluded the old individuals.^6^, ^21^, ^22^ Based on the EAU guideline, both RT and RP are all radical treatments for patients with localized PCa. However, researches about old people aged over 75 years are lacking, doctors should take the poor conditions of the elderly into account when making the decision. Based on these considerations, we attempted to assess the efficacy of RT versus RP as the primary therapy in a contemporary cohort of US men diagnosed with cT2 PCa. We observed that, for PCa people aged over 75 years, those received RP showed less likeliness to die compared with those received RT. Besides, the patients in the RP group had lower risks of CSM than their counterparts who received RT.

In one observational study targeted to Swedish PCa patients of all ages and all clinical stages, men treated with RT are more likely to develop cancer‐specific death for the low‐ and intermediate‐risk cancer.^23^ In another observational study from SEER database, men treated with RP have higher cancer‐specific‐survival rate after metastasis than those treated with RT. The difference appears more significant among intermediate‐ and high‐risk cancer. 24 Although people with the age ≥75 were included in these studies, subgroup analyses of age and clinical T stage were not performed. According to Kevin R. Rice, et al, for low risk PCa in men ≥70 years of age, no significance was found in the comparison between RP and RT.^11^ Although baseline characteristics are different in the two groups, interestingly, the results from locally weighed regression (IPTW and SMRW models) suggested variations between the two therapies had little effect on the clinical outcome.

For lack of any published or ongoing randomized controlled trial testing the choice of curing cT2 PCa for men aged over 75 years, our observational study could provide important reference to the decision‐maker. Even more noteworthy is that our comprehensive assessment overcomes the limited ability to account for unmeasured confounders contrast to previous reports.

There are still some limitations of our study. Despite our efforts to overcome the selection bias and confounders in statistical analysis to the maximum extent, there are still indicators disturbing the accuracy of our results, which is natural in any observational study. Still another limitation is the lack of data about the details of androgen deprivation therapy using, which affects the oncological outcome. Last, although we believe the treatments accord with contemporary standards, treatments may be different due to constant evolution. In particular, the surgical procedures of RP and the radiation dose of RT would be considered inadequate by current standards.

## 5 CONCLUSION

Patients over 75 years of age with cT2 stage will obtain more benefit from RP compared with RT, especially for patients with GS = 7 and younger age. These findings may facilitate counseling regarding the standard treatments for localized cT2 PCa among old people.

## CONFLICT OF INTEREST

The authors have no conflicts of interest to declare.

## References

[1] Ferlay J , Soerjomataram I , Dikshit R , et al. Cancer incidence and mortality worldwide: sources, methods and major patterns in GLOBOCAN 2012. Int J Cancer. 2015;136:E359. 25220842 10.1002/ijc.29210

[2] Jemal A , Bray F , Center MM , et al. Global cancer statistics. CA Cancer J. Clin. 2011;61:69‐90. 21296855 10.3322/caac.20107

[3] Soos G , Tsakiris I , Szanto J , et al. The prevalence of prostate carcinoma and its precursor in Hungary: an autopsy study. Eur. Urol. 2005;48:739‐744. 16203079 10.1016/j.eururo.2005.08.010

[4] Shao YH , Albertsen PC , Roberts CB , et al. profiles and treatment patterns among men diagnosed as having prostate cancer and a prostate‐specific antigen level below 4.0 ng/ml. Arch Intern Med. 2010;170:1256‐1261. 20660846 10.1001/archinternmed.2010.221 PMC3651841

[5] Kawachi MH , Bahnson RR , Barry M , et al. NCCN clinical practice guidelines in oncology: prostate cancer early detection. J Natl Compr Canc Netw. 2010;8:240‐262. 20141680 10.6004/jnccn.2010.0016

[6] Hamdy FC , Donovan JL , Lane JA , et al. 10‐Year outcomes after monitoring, surgery, or radiotherapy for localized prostate cancer. N Engl J Med. 2016;375:1415‐1424. 27626136 10.1056/NEJMoa1606220

[7] Kibel AS , Ciezki JP , Klein EA , et al. Survival among men with clinically localized prostate cancer treated with radical prostatectomy or radiation therapy in the prostate specific antigen era. J Urol. 2012;187:1259‐1265. 22335870 10.1016/j.juro.2011.11.084

[8] Zelefsky MJ , Eastham JA , Cronin AM , et al. Metastasis after radical prostatectomy or external beam radiotherapy for patients with clinically localized prostate cancer: a comparison of clinical cohorts adjusted for case mix. J Clin Oncol. 2010;28:1508‐1513. 20159826 10.1200/JCO.2009.22.2265 PMC3731893

[9] Cooperberg MR , Vickers AJ , Broering JM , Carroll PR . Comparative risk‐adjusted mortality outcomes after primary surgery, radiotherapy, or androgen‐deprivation therapy for localized prostate cancer. Cancer. 2010;116:5226‐5234. 20690197 10.1002/cncr.25456 PMC2975879

[10] Delongchamps NB , Wang CY , Chandan V , et al. Pathological characteristics of prostate cancer in elderly men. J Urol. 2009;182(3):927‐930. 19616228 10.1016/j.juro.2009.05.018

[11] Rice KR , Colombo ML , Wingate J , et al. Low risk prostate cancer in men ≥70 years old: to treat or not to treat. Urol Oncol. 2013;31:755‐760. 21872499 10.1016/j.urolonc.2011.07.004

[12] Bandini M , Pompe RS , Marchioni M , et al. Radical prostatectomy or radiotherapy reduce prostate cancer mortality in elderly patients: a population–based propensity score adjusted analysis. World J Urol. 2018;36(1):7‐13. 29063268 10.1007/s00345-017-2102-9

[13] Vatandoust S , Kichenadasse G , O'Callaghan M , et al. Localized prostate cancer in elderly men aged 80–89, findings from a population‐based registry. BJU Int. 2018;121(3):48‐54. 29603585 10.1111/bju.14228

[14] Rosenbaum PR , Rubin D . The central role of propensity score in observation studies for causal effects. Biometrika. 1983;70:41‐55.

[15] Terza JV , Basu A , Rathouz PJ . Two‐stage residual inclusion estimation: addressing endogeneity in health econometric modeling. J Health Econ. 2008;27:531‐543. 18192044 10.1016/j.jhealeco.2007.09.009 PMC2494557

[16] Cai B , Small DS , Have T . Two‐stage instrumental variable methods for estimating the causal odds ratio: analysis of bias. Stat Med. 2011;30:1809‐1824. 21495062 10.1002/sim.4241

[17] Hadley J , Yabroff KR , Barrett MJ , et al. Comparative effectiveness of prostate cancer treatments: evaluating statistical adjustments for confounding in observational data. J Natl Cancer Inst. 2010;102:1780‐1793. 20944078 10.1093/jnci/djq393 PMC2994860

[18] Hershman DL , Wright JD . Comparative effectiveness research in oncology methodology: observational data. J Clin Oncol. 2012;30:4215‐4222. 23071228 10.1200/JCO.2012.41.6701

[19] Bekelman JE , Mitra N , Handorf EA , et al. Effectiveness of androgendeprivation therapy and radiotherapy for older men with locally advanced prostate cancer. J Clin Oncol. 2015;33:716‐722. 25559808 10.1200/JCO.2014.57.2743 PMC4334776

[20] Wright JD , Huang Y , Burke WM , et al. Influence of lymphadenectomy on survival for early‐stage endometrial cancer. Obstet Gynecol. 2016;127:109‐118. 26646130 10.1097/AOG.0000000000001194 PMC4689634

[21] Andriole GL , Crawford ED , Grubb RL , et al. Mortality results from a randomized prostate‐cancer screening trial. N Engl J Med. 2009;360:1310‐1319. 19297565 10.1056/NEJMoa0810696 PMC2944770

[22] Bill‐Axelson A , Holmberg L , Garmo H , et al. Radical prostatectomy or watchful waiting in early prostate cancer. N Engl J Med. 2014;370:932‐942. 24597866 10.1056/NEJMoa1311593 PMC4118145

[23] Robinson D , Garmo H , Lissbrant IF , et al. Prostate cancer death after radiotherapy or radical prostatectomy: a nationwide population‐based observational study. Eur Urol. 2018;73(4):502‐511. 29254629 10.1016/j.eururo.2017.11.039

[24] Shao YH , Kim S , Moore DF , et al. Cancer‐specific survival after metastasis following primary radical prostatectomy compared with radiation therapy in prostate cancer patients: results of a population‐based, propensity score‐matched analysis. Eur Urol. 2014;65(4):693‐700. 23759328 10.1016/j.eururo.2013.05.023 PMC3825778

