# Supplementary material for: How to choose proper local treatment in men aged ≥75 years with cT2 localized prostate cancer?
Source: Cancer Med. 2019 May 8;8(7):3370–8. doi: 10.1002/cam4.2221 (PMC6601593; doi:10.1002/cam4.2221)
Supplement: Supplementary file 1 [file CAM4-8-3370-s001.doc]

**Supplement Table 1: Propensity score parameter list**

| the variables used in calculating the propensity matching | Age, marital status, race, PSA, GS | |
| --- | --- | --- |
| Propensity scoring algorithm | Logistic regression model | |
| C-statistical | 0.759 | |
| Matching method | Greedy matching within specified caliper distances | |
| Distance metric | 0.05 | |
| Matching ratio | (radiation therapy) 1:1 (radical prostatectomy) | |
| Use of replacement | With replacement | |
| Matching sample size | RT: 2152 cases | Total: 4304 |
|  | RP: 2152 cases |  |

**Supplement Figure 1: Propensity score matching schematic diagram**


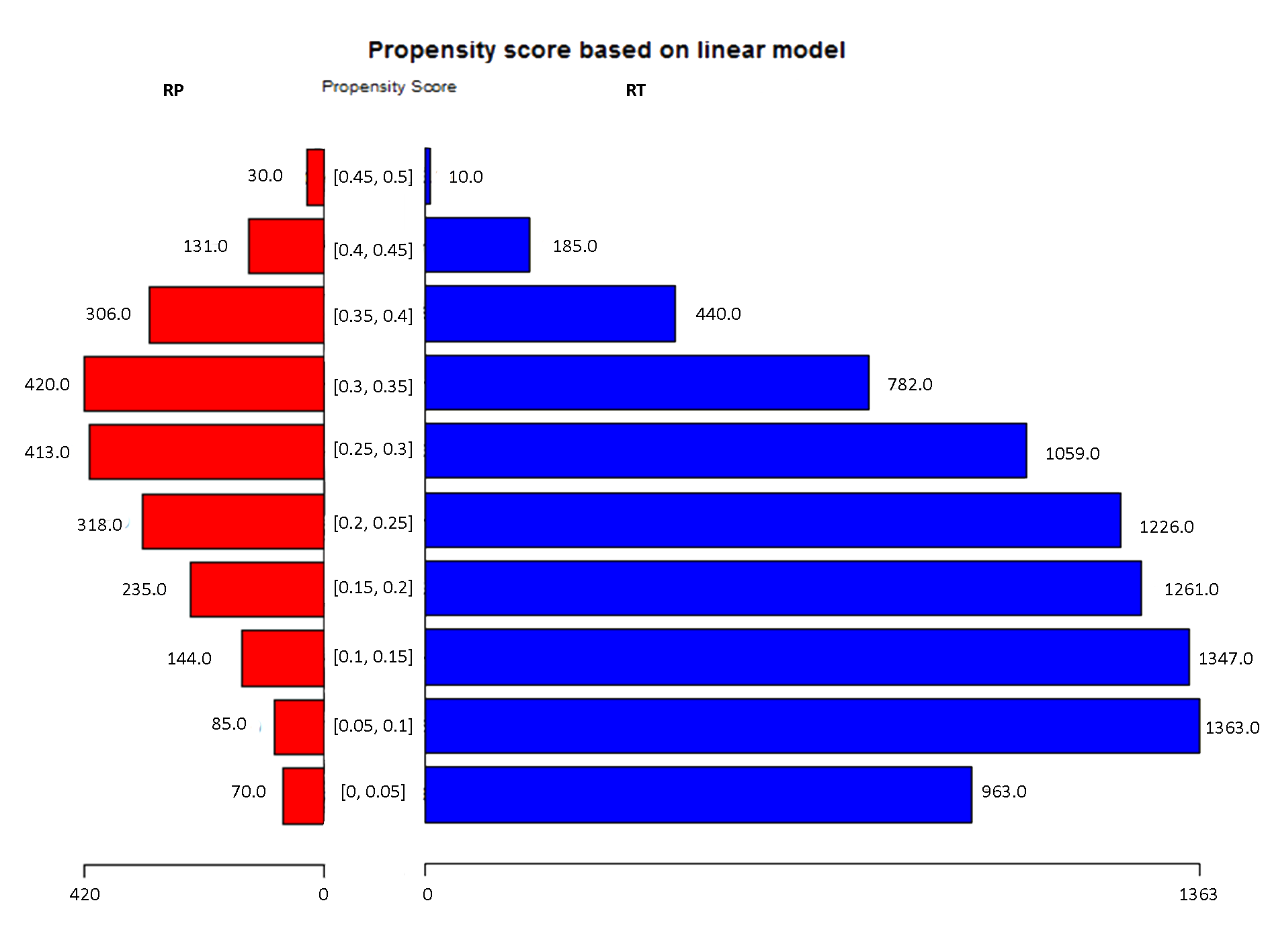


**Supplement Table 2: sensitivity analysis from propensity score matching (PSM)**

To validate the robustness of the results, the treatment effect of RT versus RP was determined using the following models:

1. Inverse probability of treatment weighting (IPTW) logistic regression model
2. Standard mortality ratio weighting (SMRW) logistic regression model
3. Covariate adjustment propensity score (CAPS) model

**Inverse probability of treatment weighting (IPTW)**

IPTW attempts to estimate the effect of treatment in the same individual [1]. It relies on the assumption that all individuals could conceivably be cared for in either RT or RP setting. After performing IPTW, the distribution of risk factors in patients received RP is equal to that found in all individuals [2, 3].

**Standard mortality ratio weighting (SMRW)**

SMRW attempts to estimate the standard effect measure that considers the exposed group as the standard population, making the distribution of risk factors in all patients is equal to that found in the RP group [4].

**Covariate adjustment propensity score (CAPS)**

After performing propensity score matching calculated by covariates, the confounders in the two treatment groups can be expressed as propensity score. Distribution of several covariates (GS and race) differs after matching (Table 2), indicating that adjustment of PS is necessary for validation.

|  | **OS** | **CSM** |
| --- | --- | --- |
| **IPTW model** |  |  |
| Non-adjusted | 0.84 (0.80, 0.89) | 0.34 (0.29, 0.40) |
| Adjusted | 0.57 (0.54, 0.61) | 0.35 (0.30, 0.42) |
| **SMRW model** |  |  |
| Non-adjusted | 0.52 (0.44, 0.61) | 0.27 (0.17, 0.42) |
| Adjusted | 0.54 (0.46, 0.63) | 0.30 (0.19, 0.48) |
| **CAPS model** |  |  |
| Non-adjusted | 0.42 (0.37, 0.48) | 0.21 (0.14, 0.32) |
| Covariate PS as continuous | 0.54 (0.47, 0.62) | 0.28 (0.18, 0.43) |
| Covariate PS as categorical  (divided into five groups) | 0.53 (0.46, 0.61) | 0.27 (0.18, 0.42) |

OS = overall survival, CSM = cancer specific mortality, PS = propensity score

IPTW model and SMRW model: adjust for age, marital status, race, prostate specific antigen, Gleason score

CAPS model: adjust for propensity score

**Supplement Table 3: statistical methods for the instrumental variable analysis**

The instrument variable (IV) for this study is the use of each therapy in different region per year (therapy cases/region/yr). For the IV to be considered valid, we performed a three-step analysis.

Step 1: to confirm that IV must be associated with the treatment.

To confirm the association between treatment and IV, in our study IV was calculated by the frequency of treatment use. Besides, it is widely considered that IV is strong with F-statistic > 10 [5, 6]. Our analysis showed that IV is highly correlated with treatment (F statistic = 911.087, p<0.001).

Step 2: to confirm that IV must not be associated with the outcome.

From multivariate analysis, IV was not correlated with OS (p=0.954) and CSM (p=0.576).

|  | **OS** | **CSM** |
| --- | --- | --- |
| **Frequency of therapy use per 10% increase** | **1.00 (0.91, 1.09) p=0.954** | **1.07 (0.85, 1.35) p=0.576** |
| **Marital status** |  |  |
| married | 1.0 | 1.0 |
| single | 1.23 (1.04, 1.46) p=0.02 | 0.73 (0.44, 1.20) p=0.21 |
| divorced/widowed | 1.34 (1.21, 1.48) p<0.001 | 1.36 (1.07, 1.71) p=0.01 |
| Unknown | 1.12 (0.97, 1.30) p=0.14 | 1.32 (0.96, 1.83) p=0.09 |
| **Age** | 1.09 (1.08, 1.10) p<0.001 | 1.07 (1.04, 1.10) p<0.001 |
| **Year of diagnosis** |  |  |
| 2004 | 1.0 | 1.0 |
| 2005 | 1.00 (0.87, 1.14) p=0.96 | 0.98 (0.73, 1.32) p=0.91 |
| 2006 | 0.99 (0.86, 1.13) p=0.84 | 0.80 (0.58, 1.11) p=0.19 |
| 2007 | 0.97 (0.84, 1.12) p=0.72 | 0.83 (0.59, 1.17) p=0.29 |
| 2008 | 0.91 (0.78, 1.07) p=0.28 | 0.79 (0.55, 1.15) p=0.22 |
| 2009 | 0.82 (0.68, 0.99) p=0.03 | 0.65 (0.42, 1.00) p=0.05 |
| 2010 | 0.75 (0.56, 0.99) p=0.04 | 0.66 (0.34, 1.28) p=0.22 |
| 2011 | 0.93 (0.70, 1.25) p=0.63 | 0.89 (0.45, 1.73) p=0.72 |
| 2012 | 0.88 (0.63, 1.23) p=0.46 | 0.73 (0.33, 1.61) p=0.43 |
| 2013 | 0.74 (0.48, 1.14) p=0.18 | 0.82 (0.30, 2.20) p=0.70 |
| 2014 | 0.63 (0.35, 1.15) p=0.13 | 1.0 (1.0, 1.0) p=0.99 |
| 2015 | 0.19 (0.03, 1.39) p=0.10 | 1.33 (0.16, 11.00) p=0.79 |
| **Race** |  |  |
| White | 1.0 | 1.0 |
| Black | 1.19 (1.03, 1.37) p=0.01 | 1.15 (0.83, 1.61) p=0.40 |
| Other | 0.74 (0.62, 0.87) p<0.001 | 0.43 (0.27, 0.68) p<0.001 |
| Unknown | 0.03 (0.00, 0.23) p<0.001 | 1.0 (1.0, 1.0) p=0.99 |
| **PSA** | 1.00 (1.00, 1.00) p<0.001 | 1.00 (1.00, 1.00) p<0.001 |
| **Region** |  |  |
| East | 1.0 | 1.0 |
| Pacific | 0.87 (0.73, 1.04) p=0.12 | 0.99 (0.62, 1.57) p=0.95 |
| North | 0.99 (0.84, 1.17) p=0.90 | 0.79 (0.50, 1.23) p=0.29 |
| Other(Alaska and Southwest) | 1.10 (0.86, 1.41) p=0.44 | 0.98 (0.49, 1.95) p=0.94 |
| **Treatment** |  |  |
| RT | 1.0 | 1.0 |
| RP | 0.54 (0.42, 0.70) p<0.001 | 0.33 (0.16, 0.69) p=0.003 |
| **Gleason score** |  |  |
| GS ≤ 6 | 1.0 | 1.0 |
| GS = 7 | 1.20 (1.07, 1.34) p=0.002 | 2.11 (1.50, 2.98) p<0.001 |
| GS ≥ 8 | 1.62 (1.44, 1.83) p<0.001 | 4.62 (3.30, 6.48) p<0.001 |
| Unknown | 1.44 (1.14, 1.80) p=0.002 | 2.86 (1.60, 5.10) p<0.001 |

Step 3: to determine whether the IV model could estimate the relationship between treatment and outcome

The Durbin-Wu-Hausman test was performed. The result indicated that the residual can affect the relationship (F statistic = 7.681, p=0.006), thus IV model could estimate the effect of RP and RT more accurately than the standard model (Cox multivariate regression) [5].

References

1 Curtis LH, Hammill BG, Eisenstein EL, Kramer JM, Anstrom KJ. Using inverse probability-weighted estimators in comparative effectiveness analyses with observational databases. Med Care. 2007;45(10 Supl 2):S103-S107.

2 Robins JM. Marginal structural models. In: 1997 Proceedings of the Section on Bayesian Statistical Science. Alexandria, VA: American Statistical Association, 1998:1–10.

3 Robins JM, Hernan MA, Brumback B. Marginal structural models and causal inference in epidemiology. Epidemiology 2000;11:550–60.

4 Sato T, Matsuyama Y. Marginal structural models as a tool for standardization. Epidemiology 2003;14:680–6.

5 Baiocchi M, Cheng J, Small DS. Instrumental variable methods for causal inference. Stat Med. 2014;33(February):2297-2340.

6 Rassen J a, Brookhart MA, Glynn RJ, Mittleman M a, Schneeweiss S. Instrumental variables II: instrumental variable application-in 25 variations, the physician prescribing preference generally was strong and reduced covariate imbalance. J Clin Epidemiol. 2009;62(12):1233-1241.
